# Solid–Basaloid Adenoid Cystic Carcinoma of the Ipsilateral Breast Remnant in Postoperative Luminal HER2-Type Breast Cancer: A Case Report

**DOI:** 10.70352/scrj.cr.25-0398

**Published:** 2025-11-06

**Authors:** Yuji Kobayashi, Kanako Miyazawa, Kayoko Shinseki, Akihiro Kushima, Masaya Takahashi, Mariko Fujibayashi

**Affiliations:** 1 Department of Surgery, Tachikawa Sougo General Hospital, Tachikawa, Tokyo, Japan; 2 Department of Pathology, Tachikawa Sougo General Hospital, Tachikawa, Tokyo, Japan

**Keywords:** solid–basaloid adenoid cystic carcinoma, adenoid cystic carcinoma, breast cancer, mastectomy

## Abstract

**INTRODUCTION:**

Solid–basaloid adenoid cystic carcinoma (SB-AdCC) is a rare and aggressive variant of AdCC of the breast. Moreover, it is an extremely rare subtype of triple-negative breast cancer, accounting for <1% of all breast cancers. We report a case of SB-AdCC.

**CASE PRESENTATION:**

Ten years ago, an 82-year-old woman underwent a partial mastectomy for the luminal HER2 (human epidermal growth factor receptor type 2)-type invasive ductal carcinoma of the left breast cancer. Ten years later, she noticed another lump in her left breast. A needle biopsy was performed, and an unclassified carcinoma was detected. Histological examination revealed that the carcinoma was of the triple-negative type. A mastectomy of the left breast and sentinel lymph node biopsy were performed. The postoperative pathological diagnosis was an SB-AdCC of the breast. The Ki-67 index was 80%, corresponding to a high-grade malignancy. She received postoperative chemotherapy with tegafur–gimeracil–oteracil potassium. At 1 year postoperatively, she is recovering well without signs of metastasis.

**CONCLUSIONS:**

We reported a case of SB-AdCC.

## Abbreviations

AdCC
: adenoid cystic carcinoma
C-AdCC
: classic adenoid cystic carcinoma
CA15-3
: cancer antigen 15-3
CEA
: carcinoembryonic antigen
CK
: cytokeratin
ER
: estrogen receptor
GATA3
: *trans*-acting T-cell-specific transcription factor
HER2
: human epidermal growth factor receptor type 2
INSM1
: insulinoma-associated protein 1
PR
: progesterone receptor
SB-AdCC
: solid–basaloid adenoid cystic carcinoma

## INTRODUCTION

SB-AdCC is a rare and aggressive variant of AdCC of the breast. Furthermore, it is an extremely rare subtype of triple-negative breast cancer, accounting for <1% of all breast cancers.^1,2)^ It is considered as a variant of the more common classic AdCC (C-AdCC), which is known to have a good prognosis; however, SB-AdCC exhibits a more aggressive clinical behavior than C-AdCC, with a higher incidence of distant metastases. Shamir et al. reported that SB-AdCC cases showed nodal metastases (22%), distant metastases (33%), and poor response to neoadjuvant chemotherapy, with no pathological complete response observed in treated patients.^3)^ A high Ki-67 index (>30%) may be an adverse prognostic factor for metastasis. Although platinum- and anthracycline-containing regimens have demonstrated some efficacy,^1)^ no standardized treatment recommendations are available owing to the rarity of the disease. Furthermore, poor response to conventional chemotherapy underscores the need for alternative treatment strategies. We report here a case of SB-AdCC.

## CASE PRESENTATION

**Fig. 1:**
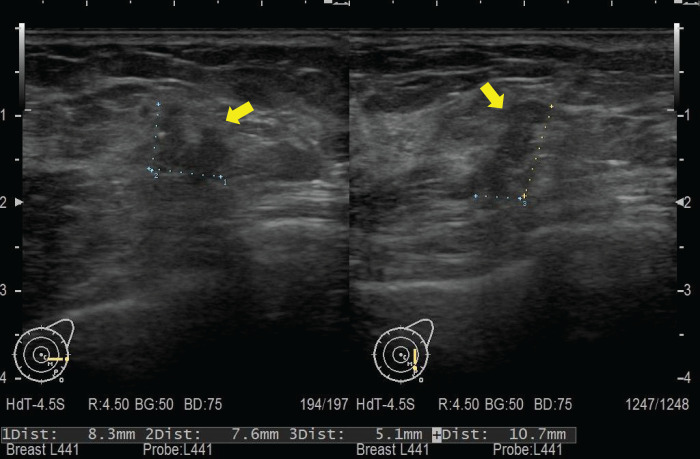
Ultrasonographic findings. Ultrasonography revealed an irregular mass in the outer region of the left breast, measuring 0.8 × 0.5 × 1.0 cm (yellow arrow). The nipple–tumor distance was 4.6 cm.

**Fig. 2:**
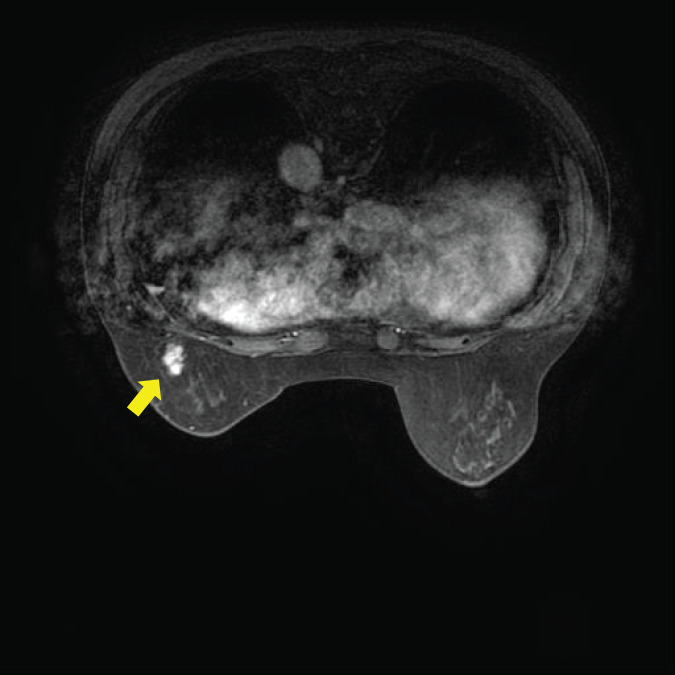
MRI findings. MRI revealed a 1.7-cm high-signal mass on DWI, which showed enhancement after contrast agent administration (yellow arrow). DWI, diffusion-weighted imaging

Ten years ago, an 82-year-old woman underwent a partial mastectomy for a luminal HER2-type breast cancer located in the left upper inner quadrant. The pathological diagnosis was T1cN0M0, Stage I, invasive ductal carcinoma. The carcinoma expressed ER and HER2 but lacked PR expression. Subsequently, radiotherapy, anastrozole, and trastuzumab were administered during the postoperative period. Ten years later, she noticed another mass in the left breast, measuring approximately 10 mm in size. The mass was located in the lower outer quadrant of the left breast and had sufficient distance from the prior surgical scar. Left breast ultrasonography findings are depicted in **Fig. 1**. A needle biopsy was performed, detecting an unclassified carcinoma, which was identified as triple-negative breast cancer via histological examination. Breast MRI revealed a 17 × 8 × 8-mm irregularly enhanced mass with T2 hyperintensity (**Fig. 2**). No metastasis was noted in the lymph node on MRI or CT. The results for tumor markers CEA and CA15-3 were negative. As the histopathological type could not be identified through biopsy, surgery was performed first.

**Fig. 3:**
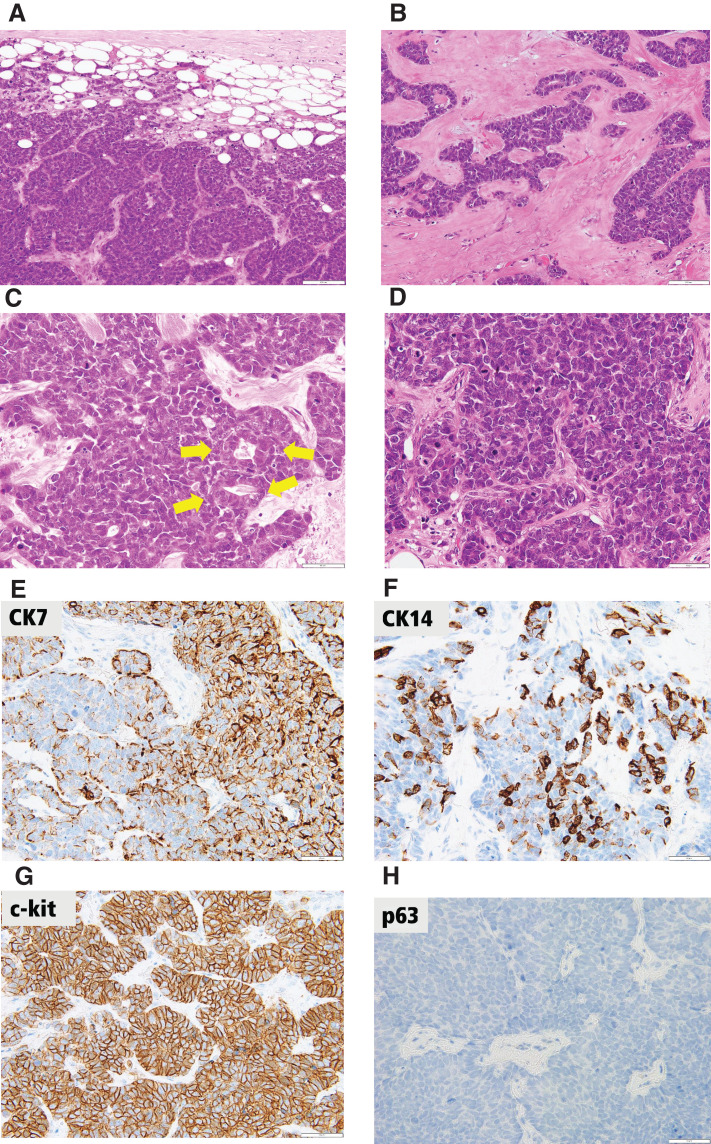
Pathological findings. (**A**) Basaloid cells composed of solid nests with geographic island patterns (H&E, original magnification ×200). (**B**) Trabecular growth pattern with myxohyaline stroma (H&E, original magnification ×200). (**C**) Glands comprising ductal epithelial cells are occasionally detected (yellow arrows) (H&E, original magnification ×400). (**D**) Basaloid neoplastic cells with hyperchromatic nuclei, inconspicuous nucleoli, and scant cytoplasm show prominent nuclear atypia and frequent mitotic figures (H&E, original magnification ×400). (**E**) CK7 expression is patchy and diffuse (original magnification ×400; scale bar at the lower right indicates 50 μm). (**F**) Basal cell marker CK14 expression is focal (original magnification ×400; scale bar at the lower right indicates 50 μm). (**G**) A luminal cell marker c-kit expresses diffusely (original magnification ×400; scale bar at the lower right indicates 50 μm). (**H**) Test result for myoepithelial cell marker p63 is negative (original magnification ×400; scale bar at the lower right indicates 50 μm). CK, cytokeratin; H&E, hematoxylin and eosin

Mastectomy and sentinel lymph node biopsy were performed. As the sentinel lymph node biopsy results were negative, lymph node dissection was omitted. She was discharged from the hospital without postoperative complications on day 6. The excised specimen revealed a lesion composed of solid nests and trabeculae of basaloid cells with high nuclear-to-cytoplasmic ratios, which provided a dark-blue appearance. Glands containing ductal epithelial cells were occasionally detected. Dense collagenous stroma and focal myxohyaline matrix were present around the basaloid nests. Immunohistochemical staining revealed triple negativity with ER and PR non-expression and HER2 non-overexpression. The Ki-67 index was very high (approximately 80%). Luminal cell markers were expressed (CK7 patchy and c-kit diffuse), and basal cell markers were focally expressed (CK5/6 and CK14). Test results for myoepithelial markers (p40 and p63), GATA3 and neuroendocrine markers (chromogranin, synaptophysin, and INSM1) were all negative. Solid components account for more than 90% of the whole tumor. These findings were generally suggestive of the diagnosis of SB-AdCC (**Fig. 3**). The pathological diagnosis was T1cN0M0, Stage I, SB-AdCC as triple-negative breast cancer.

SB-AdCC has a poor prognosis; therefore, we considered performing chemotherapy with anthracyclines or taxanes. However, owing to her advanced age, the side effects of such chemotherapy could have led to a decline in activities of daily living. Consequently, she underwent postoperative chemotherapy with tegafur–gimeracil–oteracil potassium. At 1 year postoperatively, she is recovering well without signs of metastasis.

## DISCUSSION

AdCC is a rare type of cancer with histological characteristics of a biphasic tumor composed of epithelial–myoepithelial cells and has 3 main growth patterns: cribriform, tubular, and solid.^4)^ Regarding clinical behavior, compared with other breast carcinomas, AdCC is a slow-growing tumor and generally has an indolent clinical course despite the triple-negative status.^5)^ AdCC is a low-grade tumor with a favorable prognosis and a 5-year survival rate of 80%–89%.^6)^ In contrast to the favorable features of AdCC, SB-AdCC, although an AdCC variant, exhibits unfavorable features and prognosis. Although C-AdCC has favorable 5- and 10-year disease-free survival (DFS) rates of over 95% and 90%, respectively, SB-AdCC shows significantly reduced DFS rates. A previous study reported a median DFS of only 46.5 months for SB-AdCC, compared with 151.8 months for C-AdCC.^1)^ SB-AdCC should be differentiated from small cell carcinoma, Merkel cell carcinoma of the skin, and invasive ductal carcinoma of basal-like subtype. Our case demonstrated characteristic histologic features. Immunohistochemically, the lesion displayed a triple-negative status, a high Ki-67 index, and negativity for neuroendocrine markers. For accurate diagnosis of breast AdCC, especially for SB-AdCC, molecular pathology is becoming increasingly important. To detect *MYB* activation, molecular testing for gene rearrangements, amplifications, and enhancer hijacking is recommended. Furthermore, molecular analyses of other gene abnormalities, such as NOTCH pathway gene mutations and chromatin remodeling gene mutations, are needed. Unfortunately, we were unable to perform molecular analysis or immunohistochemical staining for MYB protein at our facility.

Tegafur–gimeracil–oteracil potassium is an oral fluoropyrimidine chemotherapy drug combination that has demonstrated potential in treating recurrent triple-negative breast cancer; however, it is not a standard 1st-line treatment in new primary triple-negative breast cancer.^7)^ Inoue et al. reported that the tegafur–gimeracil–oteracil potassium combination is feasible as add-on therapy in Japanese patients with triple-negative breast cancer following neoadjuvant or adjuvant chemotherapy.^8)^ This report indicates that adding tegafur–gimeracil–oteracil to anthracycline- or taxane-based therapy as adjuvant chemotherapy resulted in prolonged relapse-free survival and overall survival. We referred to tegafur–gimeracil–oteracil add-on therapy reported by Inoue et al. This case involved an elderly patient, making it difficult to use conventional chemotherapy due to its strong side effects. However, since SB-AdCC has high malignancy, we considered administering some form of adjuvant chemotherapy. Based on the report, we decided that tegafur–gimeracil–oteracil has a partial effect. The regimen administered as adjuvant therapy was 40 mg twice daily, in the morning and evening, for 14 consecutive days, followed by 7 days of rest. The recommended dose calculated based on the body surface area for this case was 50 mg. However, due to concerns about side effects, the dosage was reduced by 1 level. Adjuvant therapy was performed for 1 year without side effects.

## CONCLUSIONS

We reported a case of SB-AdCC.

## DECLARATIONS

### Availability of data and materials

The dataset supporting the conclusions of this article is available in Springer Open.
